# A white-box approach to microarray probe response characterization: the BaFL pipeline

**DOI:** 10.1186/1471-2105-10-449

**Published:** 2009-12-29

**Authors:** Kevin J Thompson, Hrishikesh Deshmukh, Jeffrey L Solka, Jennifer W Weller

**Affiliations:** 1 Computer Science Dept, University of North Carolina at Charlotte, Charlotte, NC, 28223, USA; 2 Department of Pathology and Immunology, Washington University School of Medicine, Saint Louis, MO, 63110, USA; 3 Department of Bioinformatics and Computational Biology, George Mason University, Manassas, VA, 20110, USA; 4 Department of Bioinformatics and Genomics, University of North Carolina at Charlotte, Charlotte, NC, 28223, USA

## Abstract

**Background:**

Microarrays depend on appropriate probe design to deliver the promise of accurate genome-wide measurement. Probe design, ideally, produces a unique probe-target match with homogeneous duplex stability over the complete set of probes. Much of microarray pre-processing is concerned with adjusting for non-ideal probes that do not report target concentration accurately. Cross-hybridizing probes (non-unique), probe composition and structure, as well as platform effects such as instrument limitations, have been shown to affect the interpretation of signal. Data cleansing pipelines seldom filter specifically for these constraints, relying instead on general statistical tests to remove the most variable probes from the samples in a study. This adjusts probes contributing to ProbeSet (gene) values in a study-specific manner. We refer to the complete set of factors as biologically applied filter levels (BaFL) and have assembled an analysis pipeline for managing them consistently. The pipeline and associated experiments reported here examine the outcome of comprehensively excluding probes affected by known factors on inter-experiment target behavior consistency.

**Results:**

We present here a 'white box' probe filtering and intensity transformation protocol that incorporates currently understood factors affecting probe and target interactions; the method has been tested on data from the Affymetrix human GeneChip HG-U95Av2, using two independent datasets from studies of a complex lung adenocarcinoma phenotype. The protocol incorporates probe-specific effects from SNPs, cross-hybridization and low heteroduplex affinity, as well as effects from scanner sensitivity, sample batches, and includes simple statistical tests for identifying unresolved biological factors leading to sample variability. Subsequent to filtering for these factors, the consistency and reliability of the remaining measurements is shown to be markedly improved.

**Conclusions:**

The data cleansing protocol yields reproducible estimates of a given probe or ProbeSet's (gene's) relative expression that translates across datasets, allowing for credible cross-experiment comparisons. We provide supporting evidence for the validity of removing several large classes of probes, and for our approaches for removing outlying samples. The resulting expression profiles demonstrate consistency across the two independent datasets. Finally, we demonstrate that, given an appropriate sampling pool, the method enhances the t-test's statistical power to discriminate significantly different means over sample classes.

## Background

Microarray technologies are high through-put platforms that measure some molecular fraction of a sample [1-5]. Gene expression microarrays assay the concentration of cellular transcripts at the time samples were harvested [1]. Depending on the probe design, the technologies allow one to quantify some fraction of the active genes' transcript levels over the conditions of interest. Accurate assessment of the transcriptional activity depends on how correctly one interprets the source of a signal [6-10]. For example, several investigators have pointed out the cross-hybridization problem: many of the probes in any given design do not uniquely bind to a single part of the genome, making interpretation of any measurement arising from such a probe problematic [11,12]. Work by our group and others pointed out that probes binding where SNPs are known to occur in expression arrays can result in an altered extent of binding, depending on the alleles present, sometimes with large consequences for the interpretation of the amount of a transcript [13-16]. Our group and others have shown that internally stable structures in either the probe or the target that limit the accessibility of each to the other can materially affect the extent of signal [17-19]. The fluorescent response from the scanner or imager is not consistent over the entire response range of the microarray itself, so limits must be imposed on the signal range from which the values are analyzed (outside the linear range of the scanner, bins must be used instead of fluorescent unit values) [20-22]. It has long been known that the variation due to sample handling may be far greater than the variation due to the primary experimental variable [23], but in the absence of internal controls and general calibration standards we must resort to experiment-specific adjustments [24]. The total fluorescence per array has been previously suggested as one test of batch consistency [25], which can be represented either as the average signal per probe or signal per ProbeSet, although in neither case cited do the investigators incorporate the scanner limitation when performing the calculation. This metric reflects the labelling efficiency per molecule, but is not sensitive to sample degradation or large differences in the number of genes expressed, so we extended the metric to include the total number of responsive probes in the linear range [21,22]. As indicated by the references given for each factor, individual investigators have shown that each of these effects can have a significant impact on the outcome of an analysis, yet, to the best of our knowledge, no one has put all of them together into a simple-to-use pipeline and then tested the final effect on analysis and comparison of experiments. The impact of the factors varies according to sample characteristics that are independent of the experimental factor (i.e. probe properties and biological properties that are not correlated to the factor of interest and not subject to estimation by the experimental controls) and this type of biological/biophysical variation has created distinct dilemmas for the Microarray field: 1) across experiments, particularly across platforms, analyses lead to inconsistent outcomes and 2) significantly correlated gene lists are not reproducible in classification accuracy across datasets, or even within a datasets but across classification algorithms [9,10,26-30]. We here demonstrate that two commonly applied data normalization algorithms used in lieu of data cleansing, RMA [6] and dCHIP [31], that take a generic approach, using all probes as equally relevant reporters, interpret signal intensities of probes differently from one another, and give different results for each dataset. Changes in individual probe signal estimates translates into changes in ProbeSet values, and those changes alter where the ProbeSets cluster and the significance of expression changes [19]. However, using the complete set of cleansing filters described above, the response patterns of component probes and the aggregated value used for the ProbeSets become much more consistent, and subsequent analyses are far more robust. Hereafter the pipeline which we present is referred to as BaFL, or Biologically applied Filter Levels.

### Black box Strategies

A number of primarily statistical approaches have been applied (e.g. dCHIP, RMA, gcRMA) [6,31-33] to remove measurement variation (from sample, handling and instrumentation sources), from microarray data, that is unrelated to the experimental factor. These algorithms function as black-box techniques in that all sources of variation are merged. The probes that cross-hybridize will differ for every individual and thus the effect will be somewhat different in each experiment; a similar effect will be observed with probes sensitive to the presence of SNPs [11-13,21]. By not handling each type of factor separately it is not possible for an investigator to understand the extent to which each factor influences an experiment's results. These methods tend to augment a models sensitivity to different sources of variance in the data, as seen by the variable output of sample classification and the significant gene lists; the outcome has been that the processed data leads to good model performance within but not between experiments, using the same or different classification methods [10]. The implication is that these approaches over-train for the factors that apply in one experiment and that those factors are not consistent in their impact on the next experiment. This would be expected if some of the result is due to variables with systematic effects on a subset of particular probes, such as the occurrence of different SNP-responsive probes that will give distinct patterns in different study populations [13-16]. In order to demonstrate that the data inconsistencies are sample/population or platform dependent, an investigator needs to be able to delve into the aggregated signal and identify discordant probes and the likely causes of their behaviour, and then perform follow-up assays as needed, such as genotyping samples. A black box method does not allow the investigator to understand which particular type of secondary assay must be performed. Our approach is to identify and remove all problematic probes in a progressive manner, categorizing them as they are removed. Post- BaFL filtering yields a final set of data with very consistent responses between experiments; in addition the investigator obtains categorizations of the excluded probes, so each can be examined and probes can then be reincorporated at the discretion of the investigator. The impact of some of the factors is study dependent (e.g. SNP representation in different populations will vary), but, since our group's interest is to identify diagnostic signatures that are general rather than specific to sub-groups, our requirements in the following analyses of specific experiments are designed to find gene patterns that are robust to individual sampling and technical variation, allowing high accuracy in sample classification, whether binary or multistate [34-40].

An advantage of the multi-probe per transcript platforms is that multiple measurements are available per gene-sample, increasing confidence in the measurement [41]. Our pipeline allows the analyst to select the number of probes that must be present to define a robust ProbeSet; the pipeline default is 4. This can be set to any value deemed reasonable by the investigator: for example, on single-probe arrays, such as the Agilent 4 × 44 k arrays or Affymetrix SNP6.0 arrays, 'one' will be the only reasonable setting. An optional constraint for multi-probe arrays is that this must be exactly the same 4 probes per array in a sample class or across the experiment.

Variation arises in the sample handling steps; this is lab dependent [7,10,26,41] and is not subject to the absolute cut-offs described above. In the BaFL pipeline, statistical tests are used to compare a given chip's performance to those in the experiment as a whole, based upon the remaining probes and ProbeSets. The tests for overall similarity include: the mean signal per probe, the total number of probes, the mean signal per ProbeSet, and the total number of ProbeSets for each array in a study. The scanner manufacturer's specification for the linear detection range can be used to set lower and higher bounds for interpretable signal [22,42]; while they may be too stringent, in the absence of calibration standards and consistent controls we concur with the opinion of others that this is a reasonable approach [24]. These are parameters that may be set by the investigator, based on knowledge of the particular system used. This comparison is used to determine included and excluded samples for the subsequent analyses and comparisons.

The collection of methods has been instantiated in a software pipeline, with a database backend, that includes the following steps: upper and lower limits on intensities that reflect scanner limitations, elimination of probes with cross-hybridization potential in the target genome along with those whose target sequence no longer appears in the reference genome (some are lost as the genome is refined), elimination of probes sensitive to regions of transcripts with known SNP variations, and elimination of probes with low binding accessibility scores. This only removes known sources of variation and therefore there may still be probes affected by phenomena such as alternative splicing, degradation mechanisms, etc. [26]. The pipeline is available as source code, and can be configured for other parameter settings, methods, and filter sets (for other types of arrays). The BaFL pipeline, in conjunction with the ProbeFATE database system, allows investigators to identify potential regions of interest, for further analysis.

## Results

The following results document the effects of each stage in the BaFl cleansing process. First, we indicate the number of probes removed per filtering step and show some evidence that the intensity values from the most thermodynamically stable probes may provide an internally consistent way to assign a lower (background) detection limit. Second, we provide evidence for the validity of each step that removes probes and samples, using batch responses. Third, we provide evidence that the cleansing methodology produces consistent expression profiles across the two independent datasets and from this we obtain models of sample class correlations that yield clear latent structure that is closely replicated between ProbeSets in the two sample classes for both of the datasets. Finally, we show that, given an adequate sampling size, the power of the basic t-test is significantly enhanced when values obtained from the BaFL pipeline are used, compared to two other methods.

### Probe Filtering Output

The probe sequence-specific filters remove probes matching unidentifiable targets, probes having cross-hybridization sources, probes no longer matching targets, probes having limited duplex accessibility or stability, and those probes for which the presence of SNPs is known to be possible. These filters are consistent across all arrays; the number of probes excluded by each filter is presented in Table 1. The order in which these filters are applied does not affect the final outcome, but some probes can be removed for more than one reason, so the class into which they fall will depend on the order in which the steps are run. The position of the probe on an array is an unambiguous identifier at the probe level: this (x, y) information for the remaining probes after all of the filtering steps, is used to retrieve intensity data for aggregation, testing and modeling, and is provided as Additional file 1[19].

**Table 1:** Effects of Probe Filters

	Platform Type			
**Filter**	**GeneFocus**	**HG_U95av2**	**133A**	**133plus2**

Unidentifiable Target	2.19% (0.06%)	2.79% (1.40%)	2.17% (0.02%)	10.80% (0.0%)

SNP	5.92% (12.11%)	1.78% (3.61%)	4.14% (8.47%)	1.94% (4.36%)

Cross-hybridization	60.06% (18.39%)	60.30% (19.47%)	60.30% (18.86%)	62.15% (15.13%)

Biophysical	5.71% (3.60%)	5.16% (3.84%)	5.58% (3.46%)	13.37% (2.81%)

Percent Probes Lost as Total and (Perfect Match).

Number of probes determined to be unusable for each filtering step across several generations of the Affymetrix human gene expression array. The total number of probes in each array is the denominator, values in parentheses refer to the prefect match probes only. Each set was run independently so one does not see the subset of probes that fall into multiple categories, and the totals see to add up to more than the number of probes present. For example, eliminating probes that lack sequence information causes the Biophysical Filter to remove2.47% (2.44% PM) of the probes from the U95Av2 list, rather than 5.17%. Note the Unidentifiable target total for U95Av2 is now 0.06%, some probes have had their status revised since this analysis was first done.

**Figure 1:**
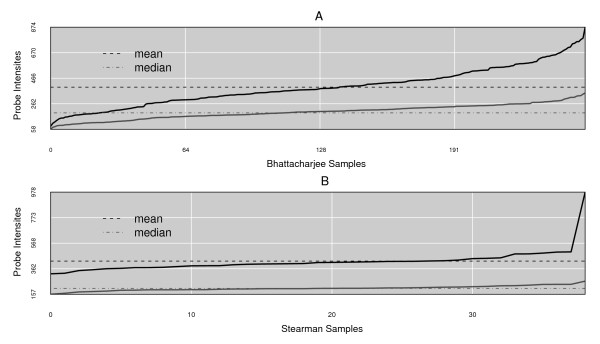
**Background Estimation**. Using the intensity (y-axis) of the set of low target-affinity probes (ΔG < -3.6 kcal/mol) over the complete set of samples in each experiment (x-axis), the median value approximates the scanner's lower limit specification: the value is 189 *f.u. *for the Bhattacharjee experiment arrays and 204 *f.u. *for the Stearman experiment arrays. The Stearman dataset has one obvious outlier (the final sample) which was detected and subsequently removed through our sample cleansing routines.

We expect that, after selecting for the minimum number of probes per gene based on the platform type, the linear range scanner limits are the parameters most likely to be changed by other investigators, depending on the type of platform and individual instrument behaviour. We have evidence that the lower detection limit can be estimated using the probes excluded with the free energy biophysical filter: these are probes that putatively cannot effectively bind target either because of probe internal structure or duplex instability. Figure 1 shows the mean (A) and median intensities (B) of this group of probes across the original set of samples: for these experiments the median is very close to the scanner cut-off that we originally chose to use; recent work on ovarian cancer data from the same platform has confirmed this feature for the low affinity probes (data not shown). Upper limits are more difficult to estimate and in the absence of calibration standards we relied on the instrument specifications.

### Filter Effects

**Figure 2:**
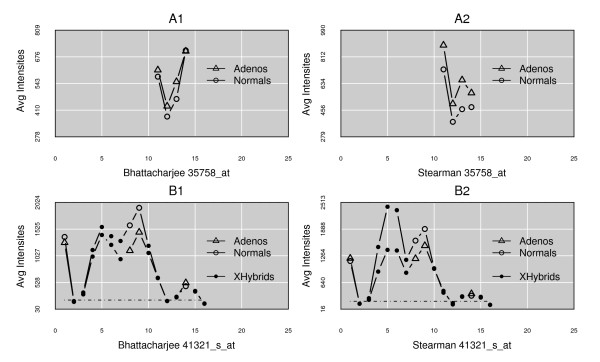
**Response patterns for cross-hybridizing probes**. The x-axis is the index of a probe within a ProbeSet; the y-axis is the mean intensity of a probe across the samples in a class. A1 and A2 show only cross-hybridizing probes for the two classes in each experiment. For a different gene, B1 and B2 show a complete set of probes, with filled circles indicating the cross-hybridizing probes. Cross-hybridizing probes are much less consistent in direction and extent of change between disease classes and across sample sets than the filtered probes (see Figure 5 for comparison).

Since some of the filters lead to a considerable loss of the usable measurement pool, an obvious question is their importance. Previously published work has shown the effect that SNPs can have on estimates of expression levels [13-16]. The largest subset of data is lost from the cross-hybridization filter. It is unknown how many of these events occur in any individual genome and how much variation can be expected across a sample population. To investigate this factor, a query was run to recover probes that are predicted to cross-hybridize (based on the consensus human genome build 36.3, identified by the ENSEMBL database) [43]. These probes were then subjected to all of the other filters, in order to isolate the impact of this factor. Examples of the results are presented in Figure 2 (A1 and A2), showing how variable the effect can be. Both the type of pattern and the level of impact differ across individual samples in unpredictable ways: it is not possible to predict particular effects *de novo*, indicating that the filter is important and should be retained (Figure 2: B1 and B2).

### Array-Batch Results

**Figure 3:**
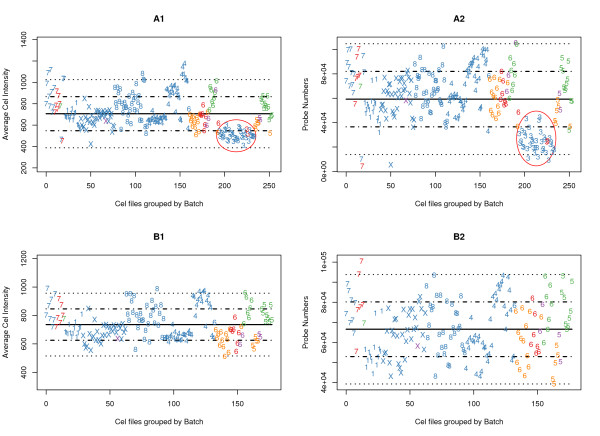
**BaFL Sample Batch Analysis**. Graphical depiction of array/batch characteristics for samples in the Bhattacharjee experiment, with array-wide mean fluorescence and mean number of contributing probes, after exclusion of probes that lie outside the scanner detection linear range. The top pair (A1 and A2) shows the complete data set while B1 and B2 show the effect of removing batch 3 and outliers. The y-axis is the mean intensity per probe (A1 and B1) or the number of probes in the linear response range (A2 and B2) and the x-axis is number of samples; batches have been clustered together and are indicated by the numeral on the graph (there are no batches 2 or 9). The mean value across the samples is shown (solid line) and the 1^st ^and 2^nd ^standard deviations from the mean are shown (dotted lines). The numeral shown indicates to which batch the sample belongs (10 is X), the color indicates disease class (blue = Adenocarcinoma, red = Normal, purple = Small Cell Carcinoma, green = Pulmonary, and orange = Squamous). The red circle emphasizes the divergent behaviour of batch 3 in both tests. The same analysis for the Stearman data is given as Additional file 2[19].

Arrays that are outliers due to sample processing problems were identified by comparing individual arrays to the batch-mean values within each experiment. Technical problems are assumed to manifest themselves by significant changes in the distribution of measurement values; the tests are described below. Figure 3A1 presents the average probe intensities per probe remaining in the cleansed array file (after removing values that fall outside the linear range) for the much larger Bhattacharjee experiment. Figure 3A2 shows the number of such probes remaining per array, with mean and standard deviation lines provided for comparison. The arrays were grouped in the plots according to their batch membership and are so labelled ('X' denotes batch 10; there is no batch 2 nor 9). Batch 3 (circled) as a whole is skewed to the lower end in both tests, so the entire set of arrays was removed from subsequent analyses. Figures 3B1 and 3B2 show the effect of removing Batch 3 from the analysis. Similar cleansing results for the Stearman experiment samples are provided as Additional file 2[19]. Since the probe sequence based filters remove exactly the same probes in all cases, the difference as to which specific probes are removed in different arrays is thus a result of the linear range filter. Batch 3 samples may have had some degradation since the arrays demonstrate both lower average probe intensity and fewer overall probes. It is reassuring to note that the Normal samples (numerals in red) were processed across several batches and do not show markedly different overall responses than the disease samples in the same batches.

**Figure 4:**
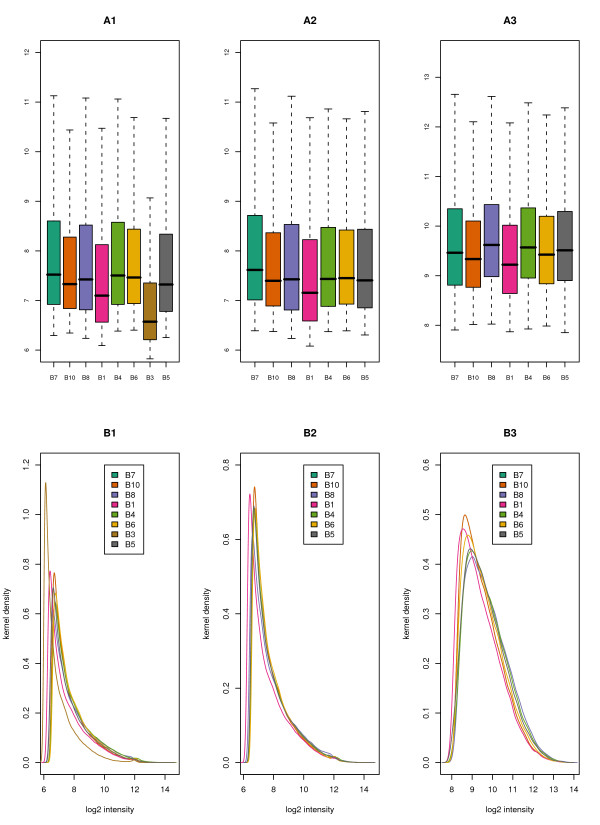
**Batch Summary of Cleansing Process**. Batch characteristics resulting from the affy package analysis. Boxplots (top row) and densities (bottom row) of the Bhattacharjee data: summary of batch intensities. The color of batch results is consistent in all graphs, as indicated in the key in the B panels. The y-axes are the log_2 _of the intensities of the probes in a batch. Panels A1 and B1 depict the completely unfiltered data set, including all probes and Batch 3: note the obvious offset in Batch 3 and the strong skew to the resulting distributions. Panels A2 and B2 show the effects of removing Batch 3 and additional outlying samples, but include all probes: the skew remains significant but no batches are outliers. Panels A3 and B3 show the output after both sample cleansing and BaFL probe filtering: the distribution is more normal but the tails have been truncated. Note that the total density scale in panel B3 is reduced relative to B1 and B2 because fewer probes are included and less variance is observed. Similar output for the Stearman experiment is provided as Additional file 3[19].

The affy package [25] results, when graphed, also indicate that there is a significant difference in Batch 3 properties; therefore this outcome is not an artefact of our probe cleansing methodology. As input to the packages graphics, the mean probe intensity across the arrays in a given batch was created as a mock CEL file (such files can also be useful for direct input into R packages; the one we created is available on the Supplementary Materials Web site for this article, see the Cleansing Results section [19]). We compared the unfiltered data, with and without the outlying samples, and the filtered data, with and without the outlying samples. Boxplots and kernel density plots for the outcomes at various data processing stages are shown in Figure 4. Batch 3 is clearly an outlier in both the boxplot and density representations in the unfiltered data (A1 and B1). The middle column of graphs (A2 and B2) show the effect of removing Batch 3 *and *outliers in other batches, but without BaFL filtering of the probe data (note that this is the data set used as input to the RMA and dCHIP algorithms [6,31] ). The data distributions still demonstrate a substantial skew. The right-most column of graphs (A3 and B3) show the effects of combined data and sample cleansing with BaFL probe filtering. Note that the linear range cut-off enforces the truncation of the distributions, which is most visible in the boxplots. The most nearly normal distribution is observed in panel B3 of Figure 4. Removal of Batch 3 accounted for 38 samples in the Bhattacharjee experiment. Similar output for the Stearman experiment is provided as Additional file 3[19].

To note a final batch anomaly, after probe filtering a localized region of persistently low-intensity spots (below the cut-off threshold) was observed within a small circle of the arrays in Batch 10, affecting an area of ~ 5,600 probes (2πr^2^; radius = 30). Array image representations were constructed by constructing mock .CEL files using the R affy package (provided on the Supplementary Materials Web site for the article, under Cleansing Results, as BaFL Cleansed Batch Summaries [19]). Since we constrained the final dataset to consist of probes common to all samples, these probes were excluded from our final set. A possible consequence is elimination of the related ProbeSet if the number of probes thereby dropped below 4. The filters did not remove these probes from the other batches, so, had we not removed them, the consequence would have been a batch-specific decrease in the apparent expression of these probes, or their related ProbeSets. Batch 10 contained no Normal samples and only one non-Adenocarcinoma sample, which could have led to the false positive discovery of these ProbeSets as significant in lung cancer Adenocarcinoma.

### Consistency of Probe Response

**Figure 5:**
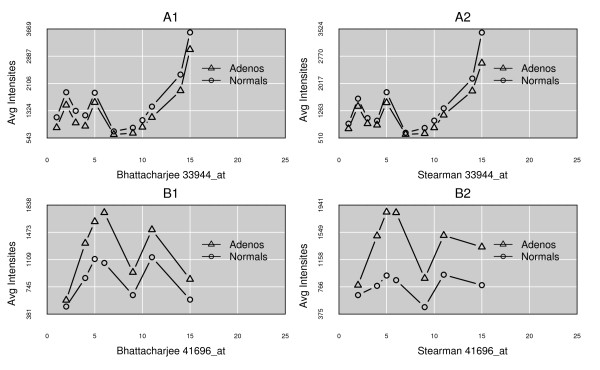
**Measurement Profile Consistency**. Measurement profile consistency for a probeset across experiments. One example is given for each of the two types of ProbeSet probe response classification categories: Significantly differentially expressed (DE) or not. Panel A1 is for a non DE ProbeSet in the Bhattacharjee experimental results and A2 is for the same ProbeSet in the Stearman experiment. B1 and B2 are for a DE ProbeSet in each experiment. Mean intensity for the probe in the sample class (fluorescent units) is on the y-axis and probe index within the given ProbeSet is given on the x-axis. Intensities are not on the same scale for the two experiments since the labelling was done independently; it is the patterns and relative intensities that are conserved.

Our goal is to find strong diagnostic signatures that are not sensitive to individual sample differences within a class. Thus, the last filtering step that we apply is to identify the intersection of common probes over the samples: the x and y locations were used to identify matched probes across all of the remaining samples. Next, sets containing at least 4 probes were collected, and from these the ProbeSet mean intensities were calculated, as the simple mean of the values of the probes remaining in the set, for each sample. Graphical displays of the average probe intensity over the samples in the class, as well as the average ProbeSet intensity over the samples in the class, show that there is remarkable consistency of the probe response profiles between experiments, some examples of which are shown in Figure 5. ProbeSet responses across samples in a class were further categorized based on the outcomes of Welch's t-tests [44], which were performed in log_2 _space with an alpha of 0.05. Each set was assigned to one of two categories: Significantly differentially expressed (DE) or not DE. Figure 5 shows a representative ProbeSet example of each type, for both experiments.

### Latent Structure Analysis

**Figure 6:**
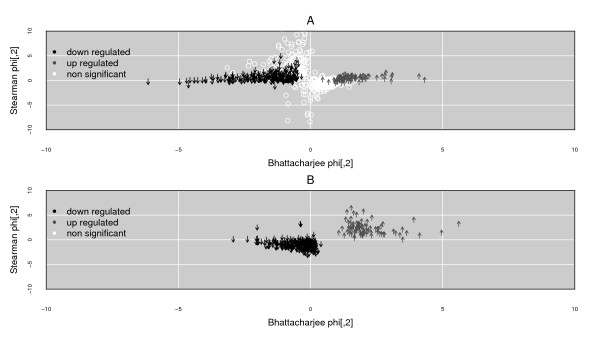
**Cross-dataset Latent ProbeSet Structure**. Cross-dataset Latent ProbeSet Structure using BaFL produced values. Two-dimensional projection calculated with spectral method of Higgs et al., as derived from the LaFon method. Sample correlation values using differential expression (DE) gene responses were used as input. Each symbol represents a ProbeSet, both color and direction of arrow indicate change: up (gray)- or down (black)-regulation, or not significantly different (white). Panel A: the 940 ProbeSets that are DE in both experiments. These ProbeSets pass BaFL pipeline criteria and are categorized as DE according to both RMA and dCHIP output (but not always in the same direction). Dark Grey upward-pointing arrows indicate up-regulated genes in the adenocarcinoma samples relative to normal samples. Black downward-pointing arrows indicate down regulation in adenocarcinoma samples relative to normal samples. Open white circles in Panel A indicate ProbeSets that BaFL does not interpret as significantly differentially expressed, while the other methods do. Bottom graph: the subset from panel A of the 325 ProbeSets predicted to be significantly DE by BaFl. The tables from which the graphs are made are provided in the Supplementary Materials Web site for the article, Latent Structure [19].

Laplacian dimensionality reduction produces an intuitive summarization of the results over the complete group of ProbeSets. Figure 6A shows the sample correlation across the BaFL values for the 940 ProbeSets that RMA and dCHIP both predict as significantly differentially expressed (by Welch's t-test). The white circles indicate those ProbeSets for which BaFl does not predict significant differential expression (at an alpha of 0.05). Figure 6B shows the 325 ProbeSets (a subset of the 940 in Figure 6A) that BaFL predicted to be DE across both experiments. The structures that are produced rotate around the x axis at 0, depending on how they correlate to the gene to which they were normalized. The spatial homogeneity of these figures demonstrates that the latent structure exists across the datasets and that this structure is not just an artefact of the BaFL selection method. The ProbeSet lists and intensity values per sample are provided in the Supplementary Materials Web site for the article, LatentStructure section, latent_data folder [19]. Graphical summaries of results based on the input from RMA and dCHIP-produced values, and with additional information about concordance in the direction of change, are available as Additional file 4[19].

### Power Analysis

**Figure 7:**
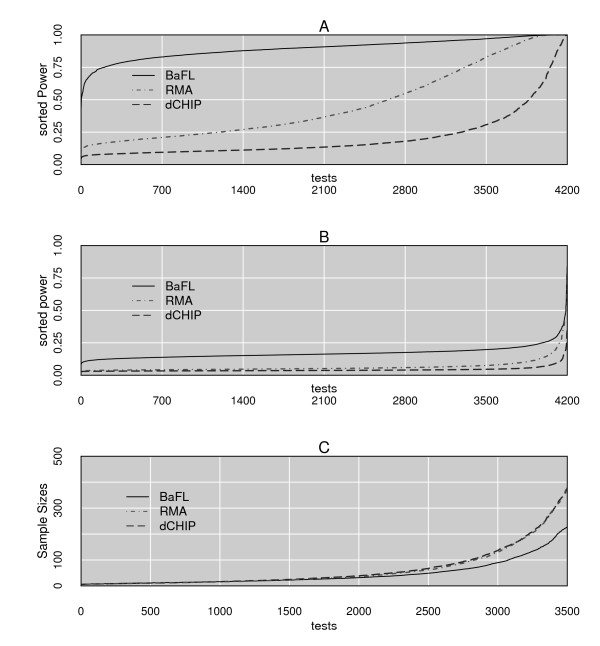
**T-test Power Analysis**. The power (sorted on the y axis) for the three probe cleansing methodologies based on t-tests of 4200 ProbeSet values per array (on the x-axis), using the same set of arrays in all cases. Panel A shows the power achieved by the Bhattacharjee adenocarcinoma sample stratification (plotted for RMA, dCHIP, and BaFl), panel B graphs the same analysis results achieved for the normal samples. Panel C presents the calculated sample size required (on the y-axis) in order to achieve a power = 0.8 (at α = 0.05) presuming the ProbeSet variation is adequately reflecting the true variation from the Stearman measurements. Although all 4200 ProbeSets were used in the simulation, the x-axis is truncated to show the beginning of the rise coming off the baseline.

Microarray technology has the well-known difficulty that N >> P, that is, far more genes are assayed (N) than samples (P) are available. Thus a large fraction of the microarray literature is dedicated to the description and validation of algorithms that normalize the data and minimize the family wise error rates of a given test, in these assays the number of genes incorrectly classified as DE. In essence, the interplay between an effect size, effect variation, sample size and type I and type II errors are compared. Figure 7A summarizes the results of the series of t-tests performed with the 4200 BaFL-passed genes, for the Bhattacharjee experiment adenocarcinoma samples, for all three of the methods, while Figure 7B similarly summarizes the results for the Bhattacharjee experiment normal samples. In panel A it is obvious that the BaFL interpretation of values produces a significant increase in the adenocarcinoma data's power and in Panel B it can be seen that it produces a respectable relative increase in power. The analysis results shown include only values produced by each method for the 4200 ProbeSets which survived the BaFL cleansing process, but using the omitted 8,425 ProbeSets also passed by RMA and dCHIP with their respective values does not improve the results for RMA or dCHIP (results not shown).

We did not observe the same increase in the t-test power for the samples from the Stearman experiment (data not shown), as is to be expected given the small sample size. Therefore, we performed a simulation to estimate the appropriate sampling size needed to achieve a power = 0.8 (for α = 0.5). The results are presented in Figure 7C. We observed that, when the effect size is small and discrimination is challenging, the rate at which the necessary sample size increases is substantially slower for the BaFL data. We note that the final 700 tests were excluded in Figure 7C, since for all methodologies the appropriate sample size increases exponentially: for these observed differences to be considered significant between the classes the dataset would have to unrealistically large for all three methods [45].

## Discussion

The BaFL pipeline as presented here enhances the ability of an analyst to obtain reliable gene expression difference information from the Affymetrix™ U95Av2 expression microarray platform, using specific, well-defined filters to remove probes that might produce confounded responses. We describe it as a white-box approach because not only is each class of filter defined, but the output is saved as tables, such that the investigator can determine why a probe was filtered out and can resurrect it at will. Very minor changes to the pipeline allow its use with other Affymetrix expression arrays; altering the requirement for multiple probes allows it to be used with any expression array, once the filters have been generated. Some of the filters are relevant to non-expression arrays, such as the cross-hybridization and probe structure filters, so the pipeline is relevant to platforms such as the Affymetrix SNP6.0 array if the SNP filtering module is removed. The ability to evaluate probe performance can facilitate the investigator's identification of transcript or genomic regions of interest, which may prove to be correlated to the phenotype of a disease state. Finally, the BaFL approach allows the investigator to identify entire ProbeSets for which one tissue state demonstrates negligible transcript concentrations in contrast to the second tissue state.

### Modified CDFs for Computational Efficiency

Since the probe characteristics are universal to an array design, one can easily construct a modified CDF that excludes particular subsets of probes. The advantage to performing this early in the analysis pipeline is that it decreases the total number of probes one must manipulate, decreasing the computational requirements for an analysis. We expect that different investigators will have preferred CDFs: for example, the cross-hybridization filter acts as a PM only filter and if a mismatch adjustment is wanted then the investigator will perform a preliminary analysis and incorporate this information into a modified CDF. An application called ArrayInitiative, to generate such CDFs, is associated with the DataFATE system (Overall and Weller, ms in preparation).

### Sample Cleansing

Sample comparisons are usually performed prior to any data assessment, which can lead to erroneous conclusions about which are the true outliers. We have presented a protocol that proceeds via measurement characteristics to perform batch analyses for technical problems, and follows up with probeset characteristics thereafter to manage biological outliers. The selection of stringency is up to the needs of the investigator: when we relaxed the sample filtering process for the Bhattacharjee adenocarcinoma versus normal samples (from 1 to 2 standard deviations of mean intensity), an additional 28 samples and approximately 400 probesets are included in the dataset (data not shown).

### Data Cleansing

Extensive cleansing of probe level microarray data is a prerequisite to any meaningful data manipulation. Probe level cleansing, as we have described here, minimizes extraneous non-experimental factor variances, such as genotypic contributions and cross-hybridization. Most importantly a successful method returns consistent cross experimental results, including strong correlations in expression profiles and class effect sizes [46].

As shown in Figure 5, data processed through the BaFL pipeline demonstrates that the desired properties of profile consistency and proportional effect size were maintained across independent experiments, indicating that there is a correlation between gene response and disease state. Two dimensional projections produced by LaFon's method [47], shown in Figure 6, using the genes predicted to be DE by RMA and dCHIP, but using values based on BaFL cleansing, showed stronger class correlations than projections of the data using the RMA and dCHIP transformed values, and these correlations were in agreement across both experiments.

### Biological Outcome

For a data mining project using BaFL pipeline data, we selected ProbeSets present in the adenocarcinoma state (minimum of 4 cleansed probes) and absent in the normal state. That is, we searched for ProbeSets that are on versus off in two conditions, rather than for significant expression differences in ProbeSets present across all samples. Although there are only a handful of such ProbeSets, osteopontin (SPP1) was identified with that analysis (data not shown): it is implicated in both lung cancer development and patient survival [48-52]. It is also straightforward to test at the individual probe level for expression inconsistencies between neighboring probes, to predict the presence of alternate splice forms (data mining results to be published separately). The datasets produced by the BaFL pipeline at the various stages are available ot the Supplementary Materials Web site for the article, in the Cleansing Results and Final Datasets folders [19].

### Power

In a complex phenotype involving destabilization of processes that drive further changes, determining the biologically significant genes is open to considerable debate. Traditional analysis methods and the majority of the classification/clustering algorithms partition samples into Euclidean space based upon the similarity of mean expression values within groups, while maximizing the group differences. Regardless of the algorithm employed, reducing the noise and excluding uninformative genes improves the separation of classes, hence the need for down selection [44,53-57]. We compared the impact that the three probe cleansing implementations had on the power of the t-test to discriminate between sample classes. We demonstrate that, given an appropriate sample size, the BaFL results give a significant increase in the power of the test statistic to correctly classify samples, relative to the other two methods, Figure 7A and 7B. Since the same n and α exist for all three methodologies, the increase in power either is due to a decrease in the standard deviation of the mean of the individual observations or because the difference between the two class means shifted as a result of using the BaFL method [44]. If one refers to Figure 4 panel A3, it can be seen that the standard deviations are diminished for the final BaFL data, relative to panel A2. At the same time, it is likely that elimination of confounding variables facilitates a more accurate estimation of the class means [17,18], enhancing the ability to distinguish shifts that do occur, as observed in Figure 5, panels A1 and A2, in which the difference in means for probes between the sample classes is statistically significant in the BaFL cases.

### Platform Enabled Analysis Flexibility

Although not the primary focus of this report, the DataFATE analysis platform (Carr and Weller, ms under revision) that we have used provides great flexibility in designing pipeline architecture, and the ProbeFATE instance makes simple the task of sub-selecting particular types of probes for detailed analysis. For example, in order to produce Figure 2 panels A1 and A2 simple SQL was used to select probes affected only by cross-hybridization, in order to highlight the difference in their response pattern compared to the highly cleansed probes.

## Conclusions

We have presented a comprehensive protocol for preparing microarray data for gene expression level analysis, using a suite of probe sequence and measurement based filters, and have shown that by so doing more reliable target measurements result, whose trends are consistent across independent experiments. While individual components of our protocol have been published elsewhere, to our knowledge the methods have not been integrated together and the overall effect assessed. Understanding contributions to a response allows researchers to have more confidence when making cross experiment data comparisons, and will facilitate our understanding of gene behaviour within a sample. We do expect that this type of analysis will only be improved with the addition of more sophisticated noise reduction methods applied to data prepared in this manner. Finally, probe based analysis is greatly simplified if carried out with a database-enabled analysis system such as ProbeFATE, which uses the probe and its related measurement as the atomic unit of observation and has been optimized for manipulations and aggregations that build specific subsets and supersets based on user-coupled criteria.

## Methods

### Hardware and Software

A system comprised of a relational database and associated analysis tools, called ProbeFATE, was used for data storage, organization and simple transformations, the contents of which then became the basis for querying for data used in specific analyses performed with Python and R scripts. ProbeFATE was first used as part of the doctoral thesis of Dr. Deshmukh, and is a specialized implementation of the DataFATE management system developed by Drs. Carr and Weller, which is described in detail elsewhere (Carr and Weller, ms in review). This instance of the ProbeFATE system was developed for PostgresSQL 8.0.3 [58] and installed on an AMD Athlon™ 64 bit dual core processor running SUSE LINUX ™ 10.0 as the operating system. Python 2.4.1 [59] scripts were developed with the psycopg2 2.0.2 [60] module to automate the cleansing process and modify the existing system. Through this module data was extracted and manipulated, and was piped for analysis in the R 2.3.1 language environment [61] via the python rpy 1.0 module [62]. Additional software included Oligoarrayaux 2.3 [63] for the calculation of probe thermodynamic values and the python MySQLdb 1.2.0 [64] module to enable querying of the public domain Ensembl MySql database [43].

### Datasets

Two independent experiments provided the datasets used in testing the effects of the filtering algorithms. Both were studies of adenocarcinoma patients in which the assays were performed using the Affymetrix HG-U95Av2 GeneChip™, so consistency of probe placement along the transcripts in the samples is assured. Using this platform, samples are assayed by 409,600 PerfectMatch and Mismatch (PM and MM) probes across 12,625 defined genes [65]. The largest, or 'Bhattacharjee', dataset http://www.genome.wi.mit.edu/MPR/lung contains measurements taken from arrays of snap-frozen lung biopsy samples. The tissues, as described by Bhattacharjee et al. [66], consisted of 17 normal and 237 diseased samples, including 51 adenocarcinoma sample replicates, with disease category assigned after histopathological examination. The diseased samples are sub-classified into 5 states: 190 adenocarcinomas, 21 squamous cell lung carcinomas, 20 pulmonary carcinomas, and 6 small-cell lung carcinomas (SCLC) [66]. From this study we used 125 of the 190 adenocarcinoma array results and 13 of the 17 normal results; the selection criteria are described below. There exists some sample replication in this final dataset, as roughly 1/3 of the final Bhattacharjee dataset arises from either normal and disease tissue coming from the same individual or two disease samples coming from the same individual. However, these replicates were generated by macro-dissection of the tissue, and some of the disease replicates appear (from annotation) to derive from distinct tumor sections. Since this was not a genotype study we did not average or drop any of these replicates, and indeed in our hands the means of probes and ProbeSets from such biological replicates show as much variation between themselves as with data from different patients. The second, 'Stearman', dataset (http://www.ncbi.nlm.nih.gov/geo/; accession number GSE2514) consists of 39 tissue samples, fully replicated, from 5 male and 5 female patients (four samples were taken from each patient: 2 normal looking that are adjacent to the tumor and 2 actual adenocarcinoma tumor samples); one of the normal samples is missing, presumably it was removed for having unacceptably high tumor content. These sample biopsies were harvested using microdissection techniques and then snap-frozen [67].

### BaFL Pipeline Components

#### Probe Filtering

The BaFL pipeline can be divided into two filtering categories, the first, 'probe sequence', category uses only the nucleotide sequence of probe and genome for determining filters, and the second category uses an aspect of the measurement (signal) as a filter. The probe sequence filters eliminate probes having attributes that confound the interpretation of the signal intensity including: cross-hybridization, loss of target sequence, SNP presence, and structural accessibility, further described below. These filters affect all samples similarly.

I. Unidentifiable Target. The CDF base table for the HG-U95Av2 arrays (Affymetrix NetAffx; http://www.affymetrix.com/analysis/downloads/data/) was queried for all 409,600 probes for which the probe sequence annotation was known. At the time of this study, there remained 11,432 probes, representing 174 genes, of unknown provenance (personal communication, Affymetrix Technical Support to H. Deshmukh), which we eliminated from further consideration[17].

II. Cross-Hybridization and Loss of Target Sequence. Probe cross hybridization is the major confounding factor affecting the interpretation of probe responses [11,12]. We have chosen to follow the Ensembl definitions of cross hybridizations, where 23/25 nucleotides must be in alignment, and we have queried ENSEMBL Biomart: http://www.ensembl.org/biomart/martview/3ee2b94e6eb250f709ffdf9474635fdf to acquire the list used to perform this filtering step. This process identifies probes that align to a single human genome region, and eliminates those which align to more than one region of the human genome and also those that don't align at all. We note that this comparison is available only for perfect match (PM) probes and therefore if mismatch (MM) probes are included in the analysis an equivalent list must be acquired and applied, or the level of filtering is not the same in the two categories of probes, and any PM/MM comparisons will have a discrepancy in the reliability of the two measurements. Most investigators no longer make use of MM values in analysis methods, nor did we do so here - from this point forward only PM probes were considered.

III. Structural Accessibility. Probe sequences were input to the OligoArrayAux software and the free energies for the most stable intramolecular species were calculated and retrieved [63,68-70]. Parameter values selected were: temperature range 41 - 43°C, 1.0 M Na^+^, and 0.0 M Mg^2+^. The average free energy for homoduplex, as well as heteroduplex, across the range of expected structures was included as probe sequence annotation data in ProbeFATE. This information can be used to filter for probes with selected levels of stability. We chose a cut-off value of ΔG < -3.6 kcal/mol as predicting the presence of an internally stable probe structure that competes significantly with target binding. In some cases numerical instability (unstable duplex, in effect leading to division by 0 for the free energy calculation) was observed in the output, and such probes were also eliminated.

IV. Presence of SNPs. Probes identified by AffyMAPSDetector as having a corresponding transcript with one or more identified SNPs in the probe-target complementary region (from dbSNP) were excluded [13]. Although the presence of the SNP within a sample may be of particular interest to a researcher, without the individual allele call for each sample these SNPs become a confounding source of variance. For example, the probe may bind strongly to the mismatch instead of, or as well as, the perfect match, and thus the PM value will not reflect the transcript concentration.

V. Measurement Reliability. The individual CEL base tables (i.e. the raw data) may be queried to determine which of the probe signal intensity values fall within a defined range, where the range indicates the limits of the scanner's ability to provide signal that can be accurately interpreted: above background and below saturation. Values above the saturation level cannot be extrapolated to a true value [21,22,71]. Although the true range is instrument-specific, in the absence of internal calibration controls that let us evaluate this limit we used the range of 200-20,000 fluorescent units suggested by [21]. An investigator may assign other limits, suggested by experience or available controls, as appropriate. This query can be performed on the reduced probe set, subsequent to the above 4 steps, or it can be performed on the entire dataset and only those probes passing both sets of requirements can be stored for additional analysis.

VI. Statistical Rigor. In these experiments our criterion was that, in a given sample, a particular ProbeSet must have a minimum of four probes remaining, after the steps described above, before a transcript-level value would be calculated (in these experiments the transcript value was the simple mean of the set of remaining probes). Probes in smaller sets were removed. A plethora of procedural choices exists from this point forward. An investigator may choose to simply enforce the minimal acceptable number of probes per ProbeSet and ignore whether the same set is present in each sample, or enforce the complete identity of probes in all samples, depending on the research question. In the results reported here, we enforced commonality of probes, aiming to examine the same subset of alternative transcripts as much as possible. Clearly, the greater the restrictions on number and commonality the smaller the final dataset will be.

In steps I-IV, the probe sequence filters are inherent probe characteristics rather than measurement characteristics and apply equally to all arrays in an experiment done on a particular platform: only the CDF (to link probe identifiers and the x, y location of the probe to information in the annotation files) and probe sequence files are required in order to flag problematic probes. Thus the order of the first four steps is irrelevant and can be set to optimize the computational efficiency. Using our data the cross hybridization filter (II) reduces the dataset most drastically, so if it is applied first the succeeding steps will be accomplished more quickly. Once steps (I)-(IV) have been completed the results are applicable to any future experiments using the same chip design and sequence files. The last two steps described above, (V) and (VI), are experiment/scanner dependent, and it is here that an investigator's design and equipment will affect what appears in the final gene list. Scanner response limits can be re-set in the code, to reflect the behaviour of individual instruments.

#### Batch and Sample Filtering

Technical steps (handling) will cause the amount of target, the labelling of that target and the effective length of the target to vary independently of the biological factors. Similarly, biological factors, such as secondary infections in cancer patients that lead to dramatic gene expression differences compared to uninfected cancer patients, may obscure the effect of the factor of interest. Technical differences tend to be seen in 'batch' effects, i.e. in groups of samples processed in parallel, while biological effects must be screened by comparing an array to the set of all arrays in its class (which may include multiple batches) [3,25,42,72]. The Bhattacharjee data set was explicitly batch annotated [66], while for the Stearman dataset the scan date was used as a proxy for batch annotation: there were 4 dates but in 2-day pairs one month apart, so our assumption is that this reflects only two technical batches. In the following discussion, both individual probe and aggregated ProbeSet values were used to compare individual array to batch and sample class trends, as follows:

I. Probes-per-Sample

a. Compare the number of probes on an array contributing to the overall intensity to the group mean, using only those probes that survived the first 6 steps of the pipeline. Arrays for which this mean exceeded ± 2 standard deviations of the group (or class) mean were excluded from further analysis.

b. Compare the mean signal per probe on an array, to the group mean. Arrays for which this value exceeded ± 2 standard deviations of the group mean were excluded from further analysis.

II. ProbeSets-per-Sample

a. Compare the number of ProbeSets on an array contributing to the overall sample intensity to the group mean, using only those probes that survived the first 6 steps of the pipeline, and for which at least 4 probes were present in the ProbeSet. Arrays for which this mean exceeded -1.5 standard deviations of the dataset mean were excluded from further analysis. The lower tail includes those samples that would most significantly limit the probesets remaining in the final dataset. This is the rationale behind the stringency of this filter and why only the lower tail was examined.

b. Compare the mean signal per ProbeSet on an array to the group mean. Arrays for which this value exceeded ± 2 standard deviations of the group (or class) mean were excluded from further analysis.

The above two levels of filter were performed in parallel, not sequentially, so there is no order of operations dependence: failing either test was sufficient to eliminate the sample from the pool. The filter in IIa is less rigorous than the others, in order to retain more samples for the final comparison, accepting that later pruning might be required.

In an independent QC test of the arrays, we performed a parallel analysis of the datasets with the R-Bioconductor affy package [25] using mock .CEL files, where probes had been aggregated by batch. The results of this widely accepted algorithm were compared with ours for both batch and sample analysis effects: that is, with and without the 'white box' probe cleansing approach. At each stage of the above-described probe filtering process graphics of the output were generated in order to monitor batch-specific behaviour.

### Latent Structure Analysis

In this set of analyses we used algorithms coded in R to calculate the Pearson correlation matrix of the ProbeSet expression values, using the BaFL values for the 940 ProbeSets that RMA and dCHIP both predict to be significantly differentially expressed in both experiments (categorized by Welch's t-test), based upon the samples, and then projected the first Laplacian dimension of each dataset against the other. Single value decomposition was performed on the correlation matrixes, and row normalization of the orthonormal (U) matrix to the first ProbeSet provides the Laplacian dimensions [47]. A second round of analysis was performed, using a subset of 325 of those ProbeSets that BaFL predicted to be DE across both experiments.

### Power Analysis

We explored the power of each univariate t-test as afforded by the way in which the three cleansing methodologies transform signal intensities, using the R function power.t.test[46,61]. The input to the function were the lists of 4,200 ProbeSets, passed by the BaFL pipeline criteria, but with values produced by each of the three algorithms, in order to produce equivalent comparisons of BaFL to the RMA and dCHIP methods. Given the unbalanced nature of the larger dataset, a power calculation was performed for each disease state, based upon an equally paired sample size, with the underlying assumption that the observed variances and differences in means are 'real' [44,46,61,73,74]. For the third part, because the small number of samples in the Stearman experiment limits the power of any statistical analysis, we performed a simulation to explore the appropriate sample size needed to achieve a power of 0.8 (for α = 0.5), with results shown in Figure 7C.

### Availability of Code and additional Supplementary Material

This code and data and associated information are freely available to everyone and can be obtained directly from the author's Web site http://webpages.uncc.edu/~kthom110/BaFL/ or by a request to the authors.

## Abbreviations

BaFL: Biologically applied Filter Level; AffyMAPSDetector: Affymetrix MicroArray Probe SNP Detector; α (alpha): 0.05 is the common Type I error; Batch: a set of microarray chips which underwent hybridizations at the same time; DE: statistically significant difference in class expression means, for the given α (0.05); FDR: False Discovery Rates; FWER: Family Wise Error Rates, Type II error; MM: single base mismatch probes; Oligoarrayaux: http://www.bioinfo.rpi.edu/applications/hybrid/OligoArrayAux.php; φ (phi): Laplacian dimension projection; PM: perfect match probes; ArrayInitiative: a script written to generate CDFs with or without excluded probes; SVD: Single Value Decomposition; x: horizontal grid placement of probes on the microarray chip; y: vertical grid placement of probes on the microarray chip.

## Authors' contributions

KT and HD contributed equally to the development and testing of the original pipeline. KT added the second data set, produced the power analysis, all of the figures and all of the Supplementary Materials, including the Web site [19]. JWW defined the pipeline steps, the tests performed, and contributed to the manuscript writing. JLS suggested several of the statistical tests and provided critically important suggestions for ensuring that the statistical tests were correctly done and the results correctly interpreted. All four authors have approved and read the final manuscript.

## Supplementary Material

Additional file 1 This file is essentially a modified cdf, providing the (x, y) information for the probes remaining on the U95Av2, after all of the probe sequence filters have been applied. This facilitates consistent treatment of data files. Click here for file

Additional file 2 Graphical depiction of the post-BaFL cleansing of the Stearman data. Samples are grouped by a scan date proxy for batch preparation and colored according to tissue classification (red = 'adenocarcinoma' and blue='non-cancerous') Click here for file

Additional file 3 Distribution summaries for the Stearman data, by scan date. From left to right the raw data, sample and batch cleansed, and BaFL processing. Top row has box plot summaries and bottom row has kernel density plots. Click here for file

Additional file 4 The latent structure that exists between the two datasets for each of the 3 probe cleansing methodologies. The 940 Probesets that were retained by the BaFL cleansing methodology and concordantly assessed as differentially expressed for the RMA and dCHIP interpretations. Click here for file
