# Supplementary figures and images for: A white-box approach to microarray probe response characterization: the BaFL pipeline
Source: BMC Bioinformatics. 2009 Dec 29;10:449. doi: 10.1186/1471-2105-10-449 (PMC2804686; doi:10.1186/1471-2105-10-449)

### Stearman Intensities

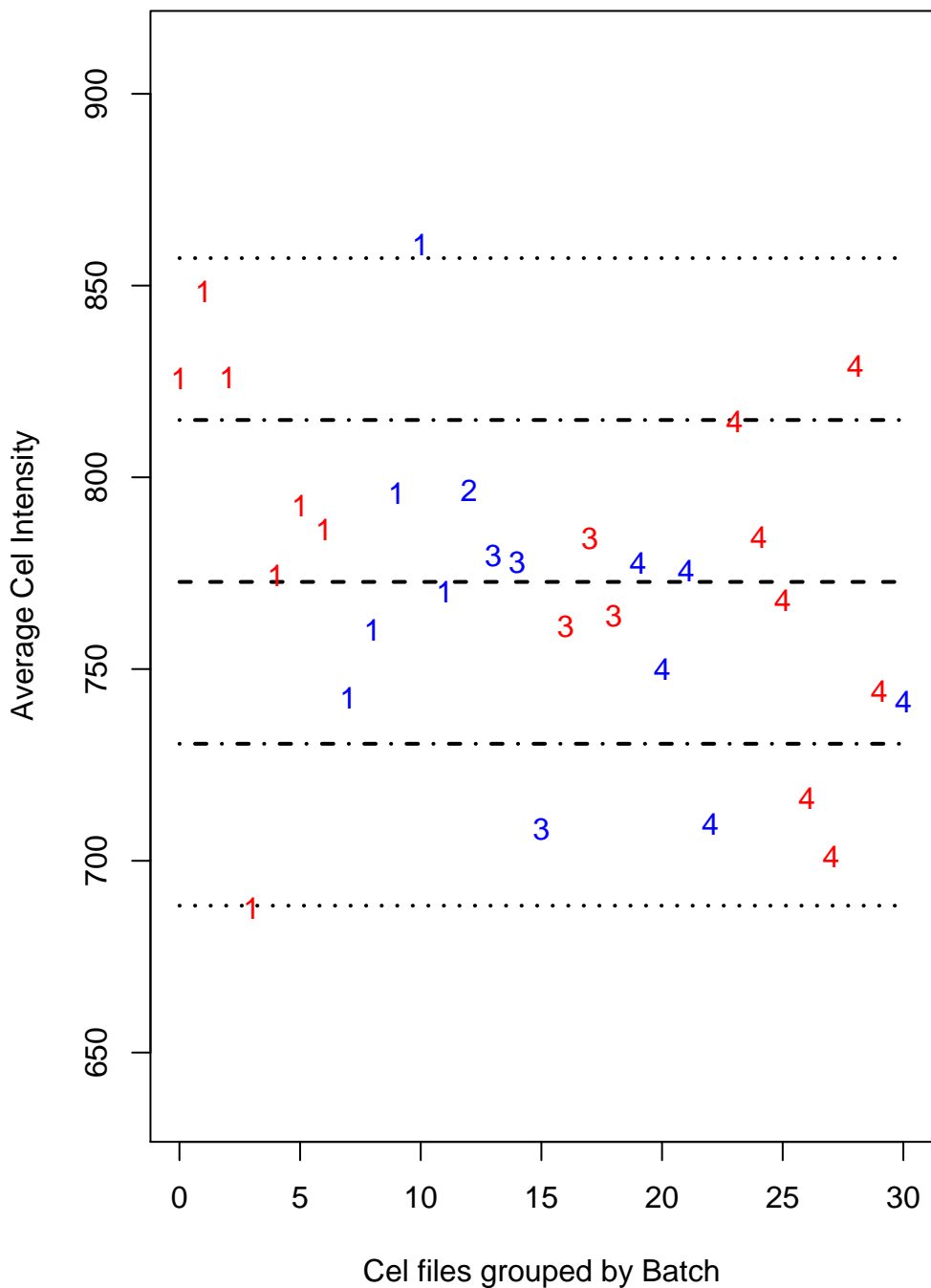

### Stearman Probe Numbers

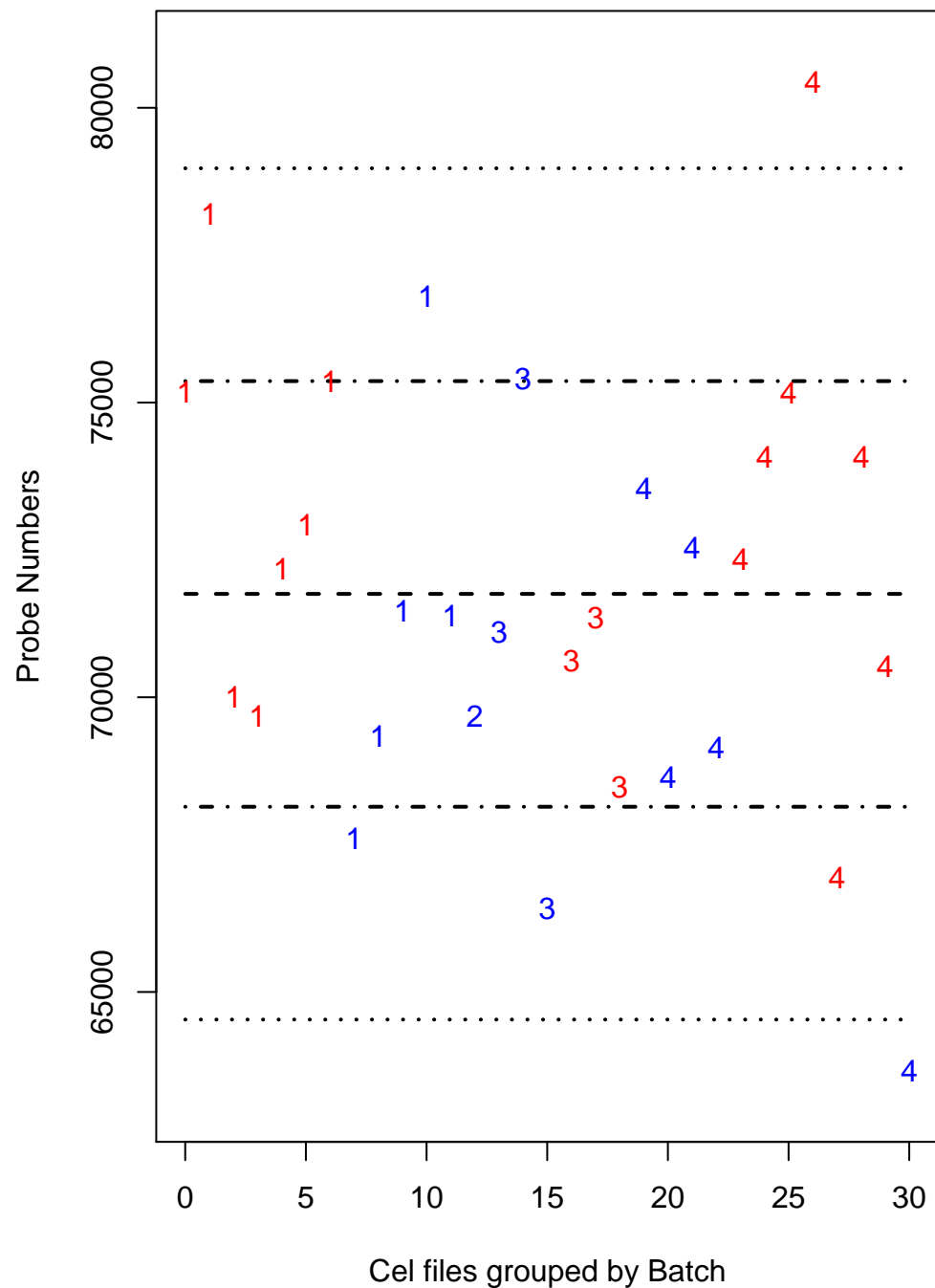

Supplement: Additional file 2 — Graphical depiction of the post-BaFL cleansing of the Stearman data. Samples are grouped by a scan date proxy for batch preparation and colored according to tissue classification (red = 'adenocarcinoma' and blue='non-cancerous') [file 1471-2105-10-449-S2.PDF]

**A1**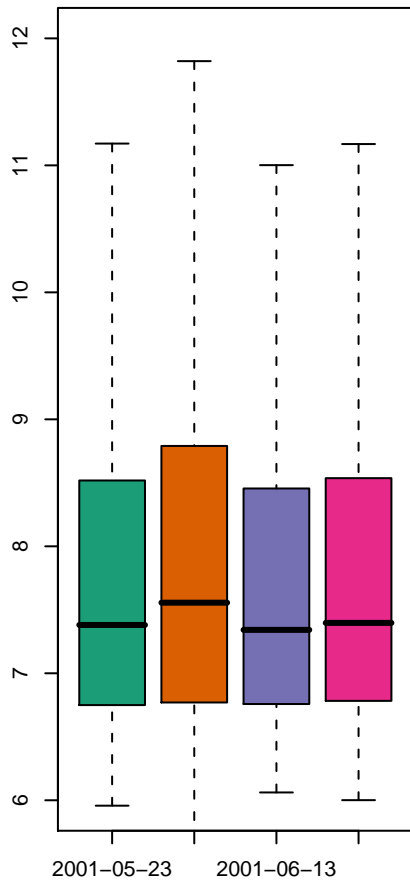**A2**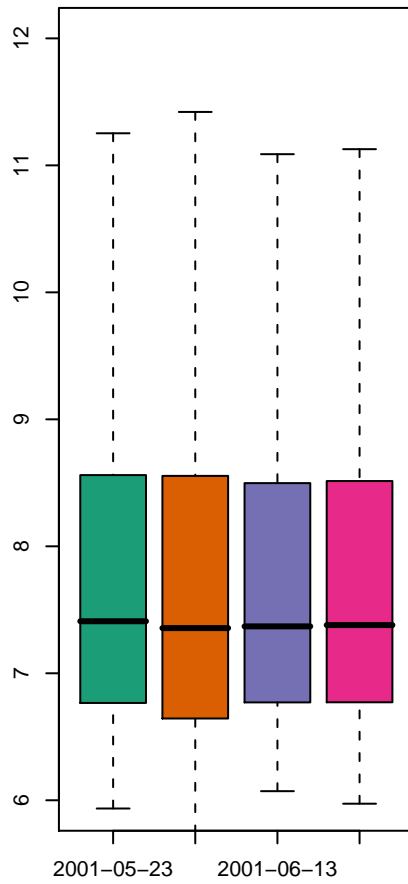**A3**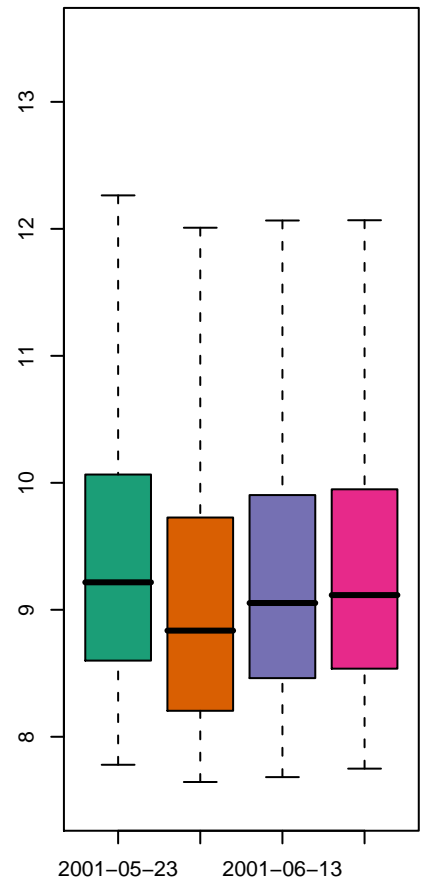**B1**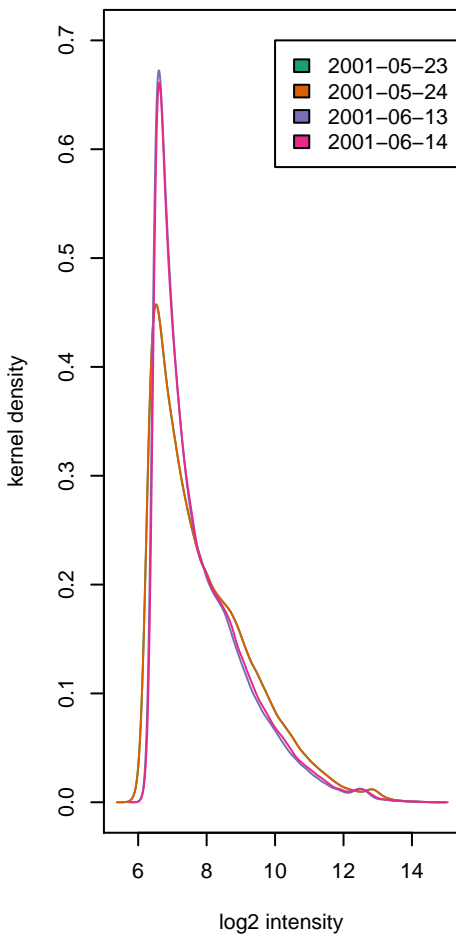**B2**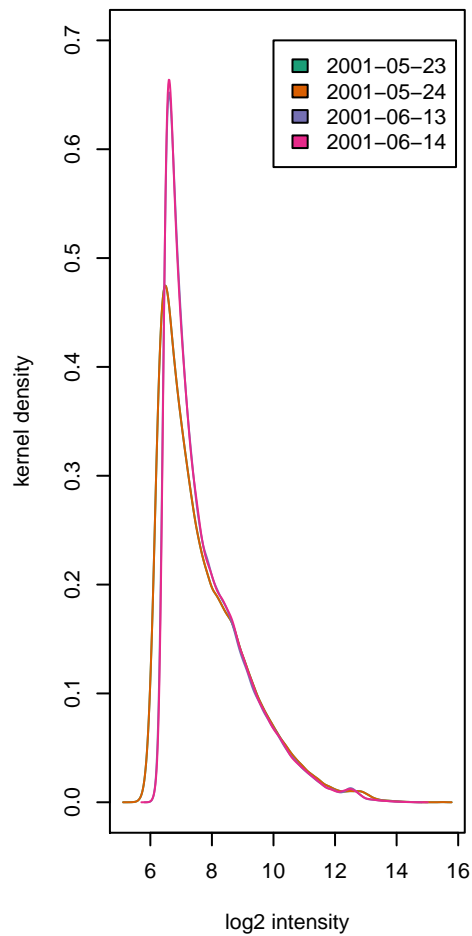**B3**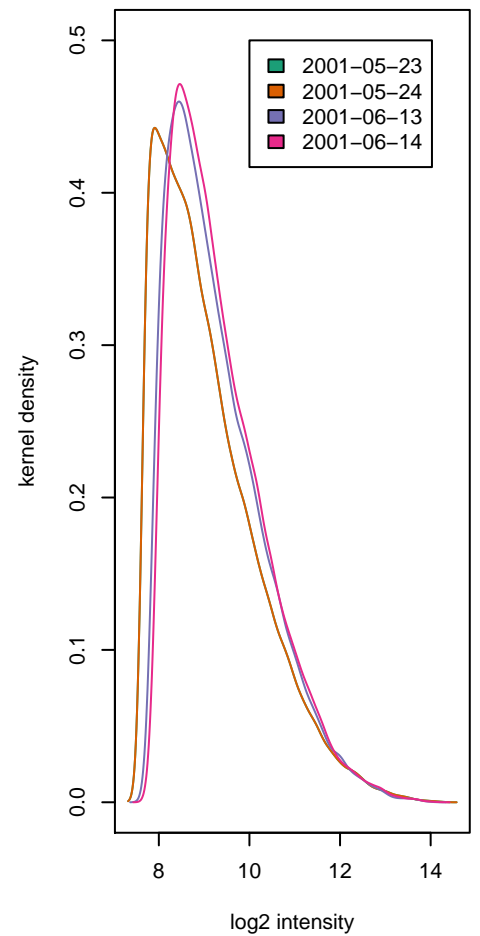

Supplement: Additional file 3 — Distribution summaries for the Stearman data, by scan date. From left to right the raw data, sample and batch cleansed, and BaFL processing. Top row has box plot summaries and bottom row has kernel density plots. [file 1471-2105-10-449-S3.PDF]
