# Supplementary material for: A white-box approach to microarray probe response characterization: the BaFL pipeline
Source: BMC Bioinformatics. 2009 Dec 29;10:449. doi: 10.1186/1471-2105-10-449 (PMC2804686; doi:10.1186/1471-2105-10-449)
Supplement: Additional file 4 — The latent structure that exists between the two datasets for each of the 3 probe cleansing methodologies. The 940 Probesets that were retained by the BaFL cleansing methodology and concordantly assessed as differentially expressed for the RMA and dCHIP interpretations. [file 1471-2105-10-449-S4.PDF]

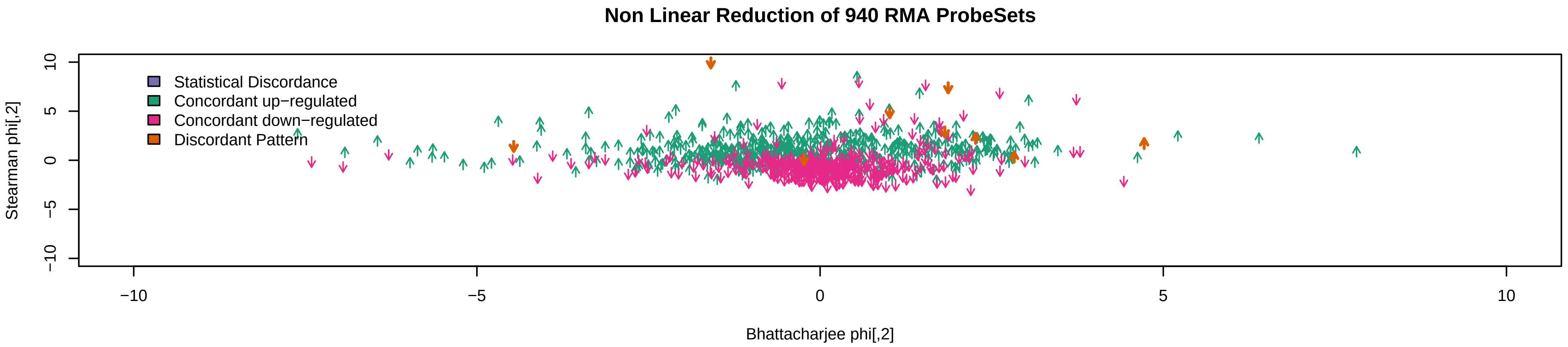
Non Linear Reduction of 940 RMA ProbeSets
Statistical Discordance
Concordant up−regulated
Concordant down−regulated
Discordant Pattern
Stearman phi[,2]
10
5
0
−5
−10
−10
−5
0
5
10
Bhattacharjee phi[,2]

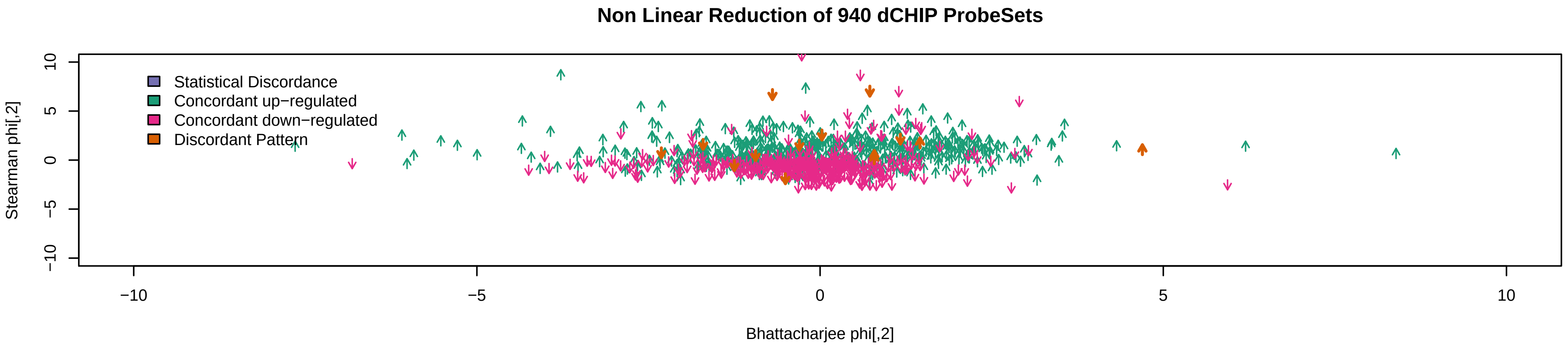
Non Linear Reduction of 940 dCHIP ProbeSets
Statistical Discordance
Concordant up−regulated
Concordant down−regulated
Discordant Pattern
Stearman phi[,2]
10
5
0
−5
−10
−10
−5
0
5
10
Bhattacharjee phi[,2]

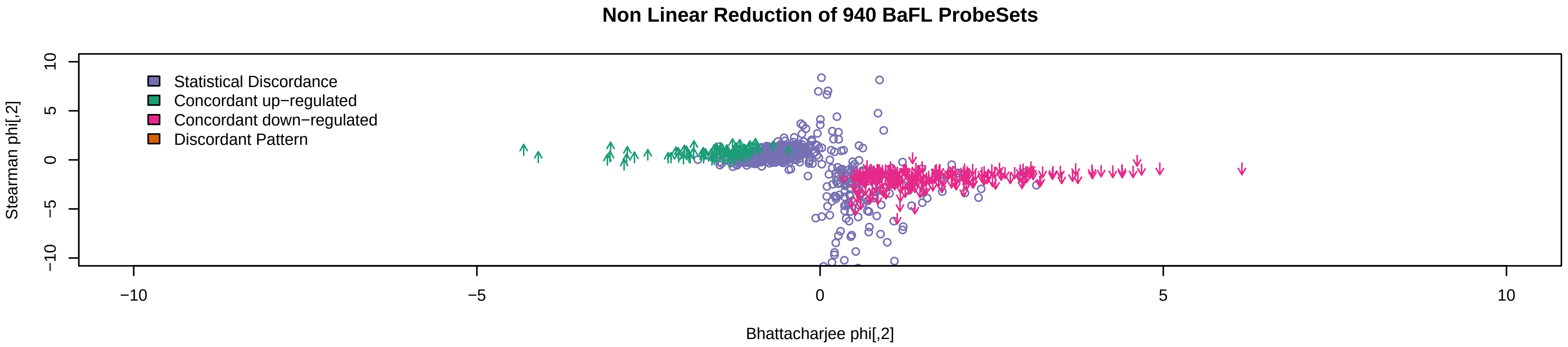
Non Linear Reduction of 940 BaFL ProbeSets
Statistical Discordance
Concordant up−regulated
Concordant down−regulated
Discordant Pattern
Stearman phi[,2]
10
5
0
−5
−10
−10
−5
0
5
10
Bhattacharjee phi[,2]
